# Immune Cell Plasticity in Inflammation: Insights into Description and Regulation of Immune Cell Phenotypes

**DOI:** 10.3390/cells11111824

**Published:** 2022-06-02

**Authors:** Andreas Margraf, Mauro Perretti

**Affiliations:** Centre for Biochemical Pharmacology, William Harvey Research Institute, Faculty of Medicine and Dentistry, Queen Mary University of London, London EC1 6MQ, UK; m.perretti@qmul.ac.uk

**Keywords:** immune phenomics, inflammation, phenotype, neutrophil, macrophage, platelet, anti-inflammatory

## Abstract

Inflammation is a life-saving immune reaction occurring in response to invading pathogens. Nonetheless, inflammation can also occur in an uncontrolled, unrestricted manner, leading to chronic disease and organ damage. Mechanisms triggering an inflammatory response, hindering such a response, or leading to its resolution are well-studied but so far insufficiently elucidated with regard to precise therapeutic interventions. Notably, as an immune reaction evolves, requirements and environments for immune cells change, and thus cellular phenotypes adapt and shift, leading to the appearance of distinct cellular subpopulations with new functional features. In this article, we aim to highlight properties of, and overarching regulatory factors involved in, the occurrence of immune cell phenotypes with a special focus on neutrophils, macrophages and platelets. Additionally, we point out implications for both diagnostics and therapeutics in inflammation research.

## 1. Introduction

Immune cells are key components of the natural defense of the human body against outside invaders, such as bacteria. The most prominent role for their involvement in pathogen defense is played by leukocytes. Even though leukocytes are crucially needed for killing and eliminating bacterial particles, excess activation of these cells can be associated with self-inflicted tissue damage and organ failure. Such overshooting immune activation is visible in sepsis patients and is associated with high mortality rates, even nowadays.

Pro-inflammatory properties of leukocytes are well-documented, whereas more recent work highlights a much more diverse immune repertoire of these cells, including: anti-inflammatory properties during models of chronic disease, as well as pro-resolution responses of cellular subsets, which need to be critically considered when assessing the impact of immune cells within the human body. Even though certain functions have been ascribed to cell populations over time (for example, efferocytosis in the case of macrophages), a cell can adapt to its requirements and its surroundings. Inflammation involves variations in plasma and organ concentrations of danger-associated molecular patterns (DAMPs) or pathogen-associated molecular patterns (PAMPs), summarized here as inflammation-associated molecular patterns (IAMPs), anti-inflammatory mediators and resolving-type mediators. All these mediators can differentially impact the cell directly and/or indirectly through the tissue-specific environment (organ). Additionally, specific peaks of plasma levels can favor production and/or mobilization of cellular subsets. However, what determines the pro- or anti-inflammatory phenotype of an immune cell? Which pathways or organ-specific environmental cues lead to cellular reprogramming or maturation? Additionally, how can this knowledge be used for diagnostic or therapeutic purposes? Before discussing underlying mechanisms, it is first important to examine how cell phenotypes can be defined and distinguished.

## 2. Techniques for Immune Cell Phenotype Assessment: A Matter of Perspective

The description of cellular properties ranges back to the very early days of microscopy, in which simple features such as color were used to discriminate a “white” or a “red” blood cell [1,2]. In recent years, research methods have become more advanced and technical, and with this, research questions have evolved to become more precise and targeted as various cellular subpopulations and phenotypic features have been exposed. Commonly used approaches for phenotypic assessment include flow cytometry and microscopy protocols, whereas lately transcriptomic techniques have also been used for identification of scarce, phenotypically distinct subclusters, even though clear international standardization measures are still missing in many areas of cellular phenotyping (Table 1).

Immune cells are frequently analyzed by flow cytometry and immunocytochemistry to define their surface characteristics [3]. This allows for the assessment of functionally relevant molecules capable of interacting with their surroundings. Furthermore, it can be used as a readout for regulative processes such as receptor internalization and shedding. Proteomics approaches are of equal importance when assessing the protein composition of a cell and showcase, for example, differences in signaling components in sterile inflammation [3], or adhesion proteins in leukocytes during atherosclerosis [4]. Aside from proteins, the lipid mediator content can also be assessed, and this mass-spectrometry-based technique was recently used to identify activation-dependent and -independent differences in lipid patterns of leukocyte subsets [5]. Many phenotypic descriptions are based on the notion of a large, overall circulating or compartment-based cellular population instead of focusing on each single cell. Recent advances in single-cell techniques yielded novel insights into cellular population and cluster-based research. Transcriptomics can be applied to study overall regulatory processes affecting gene expression on a single cell or cellular population level. Such techniques are useful in, for example, identification of spatially restricted, distinct immune cell responses, such as within the cerebrospinal fluid of multiple sclerosis patients [6]. The combination of such ‘omics’ approaches and large datasets were recently used to highlight the impact of transcription and translation within sepsis patients [7].

Assessment of physical properties can assist in identifying features which could not be revealed by the aforementioned techniques. As such, microfluidic observations together with atomic force microscopy showed that softening of leukocytes controls their dexamethasone- and catecholamine-driven demargination [8], while advanced high-throughput mechanophenotyping (termed “deformability cytometry”) was applied to differentiate active/non-active leukocytes in pleural effusions [9]. Functional assays, such as ligand-binding assays, ROS production, or migratory assays, are widely used to determine distinct responses of isolated cells to a pre-defined stimulus, which assists in identification of an activated or non-activatable cellular phenotype [10]. Most impressively, recent work by Andrés Hidalgo and colleagues elegantly showed that the integration of various data points from 4D in vivo experiments into a *behavioral landscape* profile allows for a much more holistic perspective on cellular phenotypes than a single targeted technique [11]. Therefore, cellular phenotype assessment depends on the context and required output, as well as the level of detail.

## 3. Mechanisms of Immune Cell Phenotype Switching: Outside In and Inside Out

**Figure 1:**
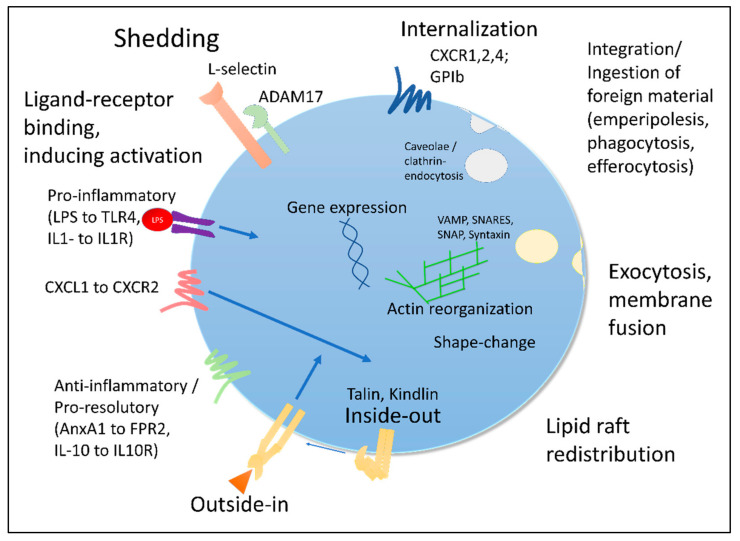
**Modes of cell phenotype switches.** Immune cells are activated by ligand–receptor binding. This may evoke different responses, including shedding or internalization of surface molecules, exocytosis and vesicle–membrane fusion, integrin activation, lipid raft redistribution, actin reorganization and shape-changes and potentially the integration or ingestion of foreign material.

Immune cell phenotype switches can be attributed to various processes, which operate in an interconnected manner (Figure 1). Generally, cellular characteristic changes will affect not only the surface composition, but equally the intracellular protein content and location, ultimately impacting the overall functionality of the immune cell.

### 3.1. Surface: Receptor Shedding, Internalization and Desensitization

Cell-surface receptor levels vary widely during inflammation based on various underlying mechanisms, including shedding and internalization. Receptor shedding requires sheddases, such as ADAMs, a class of ectodomain-shedding metalloproteinases [18]. A prominent example of receptor shedding is represented by L-selectin, which can be shed from the leukocyte surface in an iRhom2/ADAM17-dependent manner, impacting neutrophil effector functions and thus bacterial defense in vivo [19].

Receptor internalization equally occurs during inflammation/activation, impacting surface receptor levels. This process relies on endocytic mechanisms, generally involving caveolae- and/or clathrin-mediated endocytosis and redistribution mechanics [20,21]. As examples, CXCR1, 2 and 4 and GPIb have been described to undergo receptor internalization, modifying the interaction and activation potential of leukocytes and platelets, respectively, although significance for cellular functionality is context- and ligand-dependent [22,23,24]. Such events, making a receptor unavailable to its ligand or restricting signal-transduction, are also visible in the resolution-associated receptor FPR2. FPR2, like FPR1, is known to undergo desensitization, mediating excessive receptor-dependent responses, which then can be counteracted by reactivation by alternative signaling pathways, for example, by the platelet-activating-factor, allowing the cell to regain certain functionalities [25,26].

### 3.2. Regulation of Gene Expression

Inflammatory signals directly affect gene expression within immune cells. Assessment of neutrophils exposed to various stimuli identified 243 alterations in gene expression, occurring rapidly following exposure [27]. Such changes in gene expression are also detectable in leukocytes in various inflammatory diseases, such as amyotrophic lateral sclerosis, multiple sclerosis, infection, allergic reactions and rheumatoid arthritis [6,28,29,30,31]. Gene regulatory network analysis additionally highlights the effects of plasmatic sepsis-related changes on immune cell responses, affecting “hub” genes, such as STAT1, SOCS1 and ADORA3 [32]. Additionally, the (long) non-coding RNA of leukocytes is affected by endotoxemia, including GABPB-1AS1, THAP9-AS1 and SCARNA9 [33]. This showcases the sensitive regulatory interplay resulting in reprogramming and up-/downregulation of specific factors involved in immune cell functionality.

### 3.3. Activation-Dependent Vesicle Release and Membrane Fusion

Immune cells contain cargo, which can be mobilized, integrated into the membrane and/or released. An important example of exocytosis-mediated phenotype changes is the mobilization of P-selectin to the surface of platelets, which, amongst others, is affected by Ral GTPases and in a VAMP-7-dependent manner is linked to actin reorganization, subsequently resulting in platelet–leukocyte aggregate formation [34,35,36]. Additionally, in leukocytes, SNARE/NSF-dependent vesicle release will lead to reprogramming of a cell both at the surface and an intracellular level [37,38].

### 3.4. Re-Production and Re-Distribution

Sepsis evokes the extravasation of leukocytes and consumption of platelets [39,40]. Such events demand replenishing and mobilization of cellular pools to outweigh cellular deficiencies within the circulation [40]. HSCs express Toll-like receptors, which trigger myeloid differentiation and aid in replenishment of innate immune cells during infection [41]. Overall, inflammation is associated with changes in cytokine and chemokine profiles, differentially affecting myelopoiesis and cellular mobilization. As an example, IL-1 and IL-6, both of which are elevated during inflammation, are key triggers of hematopoietic stem cell proliferation and myelopoiesis [42]. Myelopoiesis and HSPC quiescence are additionally impacted by other factors, such as CXCL12 and leptin, which can be targeted by physical activity [43]. Such physical activity furthermore impacts emergency myelopoiesis, and absence or limitations of physical activity must therefore be considered in specific patient subsets, such as intensive care patients [43]. Notably, emergency myelopoiesis is regulated additionally by IL-3, affecting survival during sepsis [44], and has been linked to occurrence of neutrophil precursor cells in the circulation, visible, for example, in severe-COVID-19 patients [45]. Single-cell profiling furthermore identified shifting of progenitors to neutrophil lineage at the expense of monocytes during early sepsis [46].

In addition to novel production, organ resident cellular pools are mobilized during inflammation, such as the marginated pool of neutrophils, and also organ-specific (and possibly residing) leukocyte populations with distinct characteristics, including CD11b^hi^CD62L^lo^CXCR4^+^ neutrophils in the lung, are observed [47,48,49]. Not only leukocytes but also platelet phenotypes are affected by new production; recent work has identified a spleen-based platelet production pool which allows for the appearance of a phenotypically distinct CD40L^hi^ platelet population in sepsis [50]. This is of note, since this CD40L phenotype is associated with outcomes in pulmonary infection [51].

### 3.5. Changed Physical Properties

Changes regarding immune cell phenotypes can also occur with regard to physical properties. Generally, immune cells are migratory cells, which can change their shape and physical characteristics. Notably, in response to different stimuli, including glucocorticoids and catecholamines, leukocytes exhibit actin reorganization and a reduction in overall stiffness. This softening itself is sufficient to impact cell demargination [8]. Therefore, it is plausible and likely that both “soft” and “stiff” leukocytes co-exist within different regions of the body, representing different physical phenotypes due to their activation status and actin reorganization processes. Importantly, various pathways have been implicated in the organization and re-organization of the actin cytoskeleton, impacting circulating leukocyte numbers as well as infiltrating leukocyte subsets [52,53]. Another physical–biochemical occurrence is the redistribution of lipid raft signaling platforms [54]. Lipid rafts aid in the directed partitioning of proteins into regions of signal intensity and control clathrin-independent recycling of integrins, such as LFA-1 during chemotaxis [55]. Notably, each leukocyte class can be distinguished by specific fatty acid profiles, likely influencing lipid rafts, whereas it is unclear how these processes vary amongst distinct subclass phenotypes within immune cell populations [56]. A lipidomic study indeed revealed that fatty acid processing and lipid composition differs amongst macrophage subtypes (M1 vs. M2) with a quantified increase in exogenous fatty acid channeling into sphingolipids in M2 macrophages [57]. Thus, taking into account the various activation states and motions an immune cell undergoes, an axis-polarized phenotypic distinction (stage of recruitment, uropodia/lamellipodia formation) can emerge. Another apparent physical phenotypic distinction can be made by size. Indeed, a population of large, immature platelets has been reported to be prominently detectable in inflammatory settings of emergency cellular replenishment [58].

### 3.6. Integrin-Mediated Signaling

Integrins are bi-directional transmembrane signaling receptors of immune cells, capable of changing their activation state upon stimulation (inside-out signaling) and transmitting signals following ligand-binding (outside-in signaling) [49]. Aside from the formerly described bent-closed, extended-closed and extended-open conformations, a bent-open configuration of β2-integrins has been observed to hinder leukocyte adhesion by cis-binding of ICAM-1 [59,60]. Judging by the integrin activation states, immune cells can therefore exist in up to four different phenotypically distinct states, although these activation states appear to be of a transitional nature and to exist in parallel on leukocyte surfaces [59]. Regarding the effects of outside-in signaling on cellular phenotypes, in the leukocyte, ligand binding to an integrin can evoke a response related to effector functions, leading to a Syk-kindlin-3-dependent switch towards a ROS-productive, pro-migratory, phagocytic phenotype [61,62,63], nicely summarized elsewhere [64]. Additionally, upon phagocytic priming, macrophages and neutrophils can acquire a phenotype of pro-phagocytic responses, aiding in facilitated bacterial clearance [65]. Speaking of cellular uptake events, another peculiarity of immune cells must be taken into consideration when interpreting appearance of phenotypically distinct cellular subclasses: the uptake of foreign cellular material. Such integration of foreign material can be detrimental; for example, membrane raft incorporation of mycobacterium tuberculosis virulence factor lipoarabinomannan leads to a macrophage phenotype of impaired phagocytic capacity [66]. However, it can also promote a pro-immunogenic phenotypic priming of cells. Thus, megakaryocytes, which at first sight in the past were not considered bona fide immune cells, though our perception has changed in recent years [67], are capable of integrating neutrophil membrane fragments into newly produced platelets, thereby altering their surface composition and interaction potential [68].

## 4. Principles and Regulatory Factors Affecting Immune Phenotypes: The Environment Shapes the Cell, and the Cell Impacts Its Environment

**Figure 2:**
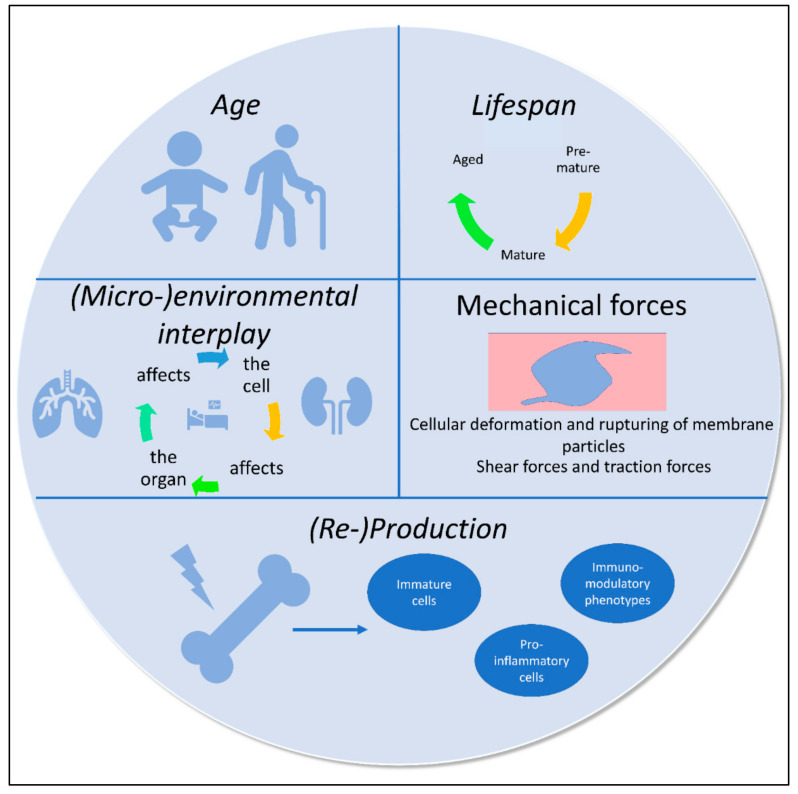
**Example of generalizable principles of immune cell phenotype changes.** Age, lifespan and microenvironments are known to impact occurrence and regulation of immune cell phenotypes. Additionally, mechanical forces, leading to deformation and disruption of membrane components, can lead to changes in shape, membrane composition, activation patterns and overall phenotype appearance. Demand-dependent (re-)production leads to occurrence of various distinct phenotypes, including immature, pro-inflammatory and immunomodulatory cellular phenotypes.

Phenotypic reprogramming of immune cells occurs due to various generalizable principles, which affect, in different ways, distribution, production and activation mechanisms within the body (Figure 2). All these regulatory processes occur simultaneously to a varying degree, leading to temporary dominance of distinct governing factors and phenotypic landscape composition.

### 4.1. Circadian Rhythms

The time of day leads to variations in leukocyte populations both in numbers and phenotypes with direct consequences for combatting invading pathogens [69]. Thus, the myeloid-specific brain and muscle Arntl-like protein 1 (Bmal1)-dependent control of inflammatory monocyte circadian rhythmicity is known to impact immune defense [70]. Tissue-specific circadian modulations of adhesion molecules and chemokines [71], together with cell autonomous oscillations, further promote organ selectivity and circadian rhythmicity of recruitment of distinct leukocyte subsets [72,73,74,75]. Additionally, plasmatic concentrations of specialized pro-resolving mediators, such as RvD_n3 DPA_, are diurnally regulated, thereby affecting both neutrophil and platelet activation [76].

### 4.2. Aging

The host, both as a whole, and each cell will mature and age throughout their lifespan, implicating differential cell-intrinsic and -extrinsic phenotypic modulators. Similar to extensive microenvironmental changes occurring in the developing organism, in which leukocytes with an impaired functional phenotype are observable [77], host ageing is equally associated with overall changes in the inflammatory milieu. Notably, increased basal levels of circulating pro-inflammatory mediators, reduced cellular regeneration potential due to double-stranded DNA breaks, as well as differentially modulated inflammatory responses of immune cells, including reduced neutrophil chemotaxis and peroxide production and macrophage cytokine production, can be observed during ageing [78,79]. Additionally, organ-specific phenotypic reprogramming occurs during ageing, implicating interaction of reverse-transmigrated neutrophils within the lung, which primes these cells for an activated phenotype responsible for the induction of remote organ damage [80]. Of note, ageing is also associated with disturbances in circadian rhythms, leading to imbalances in immune cell responses [81], and likewise, disturbed circadian rhythmicity due to genetic targeting of BMAL1 leads to premature ageing [82]. As for the cell-intrinsic maturation and ageing, aged neutrophils feature high CXCR4 expression, which mediates their clearance within the bone marrow [40,49]. In contrast to the notion of an aged cell as an exhausted “retired” cell, these are actually well-educated, “trained” and easily activatable cells, aiding in the immediate immune defense response [83,84]. The occurrence of these CXCR4^hi^-aged-neutrophils is regulated by microbiota-TLR-MyD88 signaling [83]. Nonetheless, as with most overtly active cellular phenotypes, a downside must also be considered, since persistence of these aged intravascular neutrophils is associated with thrombo-inflammation and reduced survival following myocardial infarction [85].

### 4.3. Stress

Acute and chronic stress have divergent effects on immune cell functionality; chronic stress is regarded to mainly evoke an immunosuppressive response, whereas acute stress can lead to immunostimulation [86,87,88]. Animal experiments using application of restrainer-mediated psychological stress thus led to increased leukocyte recruitment into a site of injury, showcasing modulated systemic factors and impacting phenotypic activation-dependent immune cell responses [89]. Similarly, changes in leukocyte subsets can be detected in humans when exposed to stress [90]. These responses are not surprising, in view of the hormonal changes occurring in response to stressors, including catecholamine release and glucocorticoids, and the variety of counter receptors expressed by immune cells. Nonetheless, immunologic responses following catecholamine stimulation are much more complex, indicating the dose and context dependency of leukocyte subsets, as discussed by Scheiermann et al. and others [91,92].

### 4.4. Environment/Compartment (Organ)

Each tissue/organ within the body features proprietary interaction and signaling modalities [49]. Therefore, the lung primes a noxious, overtly active reverse-transmigrated neutrophil phenotype, subsequently leading to secondary organ injury [80]. Similar observations of detectability and phenotypically distinct priming of reverse-transmigrated neutrophils with delayed apoptosis have been made, for example, in chronic inflammatory arthritis patients, highlighting either a potential reverse migration of neutrophils from the joint back into the blood stream or the return of neutrophils from other secondary injured organs to the circulation [93]. Overall, inflammatory-site-invading neutrophils take on modified transcriptional activities, involving neutrophil polarization and changing their phenotype in a site- and stimulus-specific manner [94]. As an example, in resolving inflammation settings, migrated neutrophils upregulate the pro-resolving protein annexin A1, and this could be part of resolution circuits in experimental peritonitis [95], sepsis [96] and colitis [97]. This is of special interest when taking into consideration that formation of membrane blebs, as has been described in neutrophils during apoptosis and compression-microenvironment-dependent membrane protrusion [98,99], is affected by Annexin A1 itself, regulating cell lysis and potentially membrane repair mechanisms [100]. Additionally, it is emerging that aside from traditional organ-resident cell populations, an inflammation-mediated imprinting of resident macrophages via modulated niche mechanisms leads to prolonged phenotypic re-composition of immune cell landscapes within organs [101].

### 4.5. Mechanical Forces

Immune cells exist not only within tissue compartments but also within the circulation. Circulating immune cells are constantly impacted by shear forces, leading to deformation and cell–cell collisions. Thus, taking into consideration margination and deformation capacities of immune cells, various force-adapted cellular phenotypes co-exist within the circulation in parallel. According to Simon et al.’s considerations of the modified Smoluchowski’s two-body collision theory, collisions and aggregate formation of neutrophils do occur in a shear-dependent manner, emphasizing that different immune cell phenotypic characteristics can be found within varying vascular beds [102]. Shear additionally impacts the biomechanical re-composition of leukocytes. Herein, pseudopod formation and leukocyte spreading occurs in a potassium- and calcium-dependent, shear-controlled manner with reduced shear favoring pseudopod projection [103]. Nonetheless, migration and podosome formation varies according to leukocyte and macrophage subsets [104]. Interestingly, leukocytes themselves also show a force-dependent migratory plasticity, in that they hibernate between adhesion-receptor-mediated responses of force transmission and receptor-independent nuclear-positioning-facilitated locomotion, as suggested by Renkawitz and Sixt [105,106].

Importantly, inflammation can lead to redistribution characteristics due to changed blood flow properties, involving vasodilation/vasoconstriction and endothelial damage, impacting the site- and subset-specific trafficking of immune cells in an activation- and shear-dependent manner [107,108]. Leukocytes are recruited in a simplified cascade-like manner, involving rolling, adhesion and extravasation. During rolling, sling formation and rupturing of slings can occur, which can conclusively lead to leukocyte subsets lacking certain membrane elements/particles [109]. Likewise, similar to the changed flow properties during sepsis, the introduction of artificial surfaces, for example, during renal replacement therapy or due to assist devices, also poses an environment for differential targeting and activation of leukocytes. Neutrophil activation and microparticle formation thus can be commonly observed in cardiopulmonary bypass and ventricular assist devices, as shear-dependent mechanical activation and phenotype switching appear to occur in a guanine-nucleotide-exchange-factor-dependent manner [110].

### 4.6. Inflammation and Injury—A Combination of Extremes

An inflammatory reaction in response to an injury or infection leads to disturbance and modification of several of these generalizable processes. Increased release of stress signals (catecholamines and glucocorticoids), together with disruption of circadian rhythms as well as cell consumption, are only part of the overarching modulatory environment during inflammation. Most prominently, increased levels of cytokines, chemokines, PAMPs and DAMPs lead to a complete re-composition of the immunologic landscape within the body [111].

## 5. Macrophage Phenotypes and Plasticity

Macrophages can be categorized by their localization (pulmonary, liver, interstitial, alveolar), function (pro-inflammatory, pro-resolution), characteristics (expression, cytokine release) or ontogeny (resident vs. monocyte-derived recruited). Each categorial variable is differentially impacted by inflammation, as, for example, pulmonary inflammation leads to changes in overall tissue resident monocyte/macrophage numbers and phenotypic predominance [112].

### 5.1. M1, M2 and Beyond

One of the most commonly used phenotypic distinctions involves the categorial description of M1 and M2 macrophages, implying a pro-inflammatory or anti-inflammatory/pro-resolution phenotype (Table 2) [113]. As described in the nomenclature perspective paper by Murray et al., these populations feature distinct activation patterns and can be obtained by differential activation in vitro, implicating STAT1 and STAT6, respectively [114]. Classically activated M1 macrophages are described as phagocytic, proinflammatory cytokine-releasing (IL-1, IL-6, TNF) macrophages, which can be obtained in vitro by LPS and interferon stimulation [114,115,116]. The phenotypic shifting towards M1 involves key pro-inflammatory triggers, such as TLR4, MyD88, IKK, NF-kB and IRF5, as well as JNK [117,118,119]. Notably, shifting of macrophages to an M1 phenotype favors a snowball response in that the release of proinflammatory mediators encourages the polarization of additional macrophages towards an M1 profile [120]. Alternatively activated M2 macrophages, on the other hand, can be obtained by IL-4, IL-10 and/or TGFβ stimulation and showcase subclass-specific production of IL-10, CCL22 and CCL24 [121]. Further subset categorization is used as M2a (wound-healing, CD206^hi^), M2b (regulatory, CD86^hi^), M2c (acquired deactivation, MerTK^hi^) and M2d (tumor-associated, VEGF^hi^) [114,122]. In septic baboons, a purely M1 phenotypic profile of circulating monocytes, and speculatively also macrophages, were associated with reduced survival compared to a mixed M1/M2 phenotypic profile [123]. It appears reasonable that during an acute infection an inflammatory response is required in order to clear invading pathogens; subsequently, such inflammation needs to be resolved and cleared in order to ensure organ homeostasis [124]. Aside from signaling mediators such as PI3K/Akt being involved in balancing M1/M2 profiles, NOTCH signaling (favoring M1 macrophage plasticity) and KLF4 signaling (increased in M2 macrophages) have also been reported to impact macrophage plasticity and their pro-/anti-inflammatory responses [125,126]. An M1 macrophage profile is also present during acute kidney injury and can be targeted by modulation of heparin-binding protein expression or the targeting of CLEC4e/Mincle-Syk selectively on macrophages [127,128]. Although locally, such an M1 profile is needed to combat invading pathogens, a systemic overactive M1-polarized macrophage profile is associated with deteriorated sepsis outcomes [129], whereas an M2 profile is required for resolution dynamics following an acute insult. Nonetheless, preponderance of M2-like macrophage phenotypes could hold risks of immunosuppression and thus of secondary infection. Such a post-sepsis M2-like phenotype, implicating immunosuppression, is regulated via p21, affecting p65-p50 and p50-p50-NFkB pathways within the macrophages [130]. Indeed, an incomplete M2-type conversion was hypothesized to be involved in post-sepsis immunosuppression, although this study leaves various questions still unanswered [131]. Macrophage phenotypes are not only modulated by underlying conditions or self-sustaining pro-inflammatory feedback loops; cellular interplay controls modulation of macrophage plasticity. Indeed, recent work highlights that during pulmonary infection, platelet–Treg interplay differentially primes the M2-like resolution responses in alveolar macrophages in post-pneumonic mice [51]. Nonetheless, these rather basal phenotypic attributions of M1/M2 profiles insufficiently describe the whole magnitude of cellular complexity present within the organism, since in vivo studies highlight presence of intermediate phenotypes and much more complex signaling environments [132]. Phenotypic switching of macrophages is not only impacted by pro-inflammatory mediators, but also by specialized pro-resolving mediators (SPMs). These mediators, such as Resolvin D2, are capable of controlling sepsis severity and protectin D1/neuroprotectin D1 offers protection in an influenza virus model, showing the diversity of these modulators [133,134,135]. Notably, the resolution-implicated GPCR GPR37, expressed on macrophages, controls neuroprotectin D1-mediated phagocytosis and protection against sepsis [136,137]. Thus, macrophage phenotypic changes are not only impacted by the pro-inflammatory environment, but equally by the presence of pro-resolution factors, aiding in the containment of infection and overshooting immune responses.

### 5.2. Tissue-Specific Macrophages

Aside from a purely functional connotation, localization and origin must also be taken into consideration when interpreting the magnitude of macrophage phenotypes during inflammation. Indeed, tissue origin and ontogenic programming of macrophages seem to partially outweigh the stimulatory responses during inflammation. More precisely, in a co-infection model of Heligmosomoides polygyrus bakeri and Salmonella enterica, F4/80^hi^-resident macrophages are primed for recruitment of neutrophils by release of neutrophil-attracting and -activating chemokines, such as CXCL1 and CXCL2, compared to F4/80^lo^-recruited macrophages [138].

Generally, organ-residing macrophages can be described based on their compartmentalization or cell surface markers. In the lung, the basic distinction involves alveolar- and tissue-resident macrophages, characterized by absence/lower expression of CD11b, presence of CD11c, absence of CD86 and presence of SiglecF on the surface of alveolar macrophages compared to interstitial macrophages [139]. scRNAseq techniques have aided in assessing more diverse subtypes of tissue-specific macrophages. Herein, a pulmonary SiglecF-positive (SiglecF^+^ CX3CR1^+^ MHCII^hi^) interstitial macrophage was identified, causing pro-fibrotic changes within the lung in a bleomycin model and delineating an intermediate phenotype in-between classically referenced interstitial and alveolar macrophages [140]. In the spleen, marginal metallophilic (Siglec1^+^, CD169^+^), marginal zone (SIGNR1^+^ MARCO^+^) and red pulp (F4/80^hi^CD68^+^ CD11b^lo^) macrophages can be distinguished [141]. Taking advantage of their spatial proximity, splenic CD169^+^ metallophilic macrophages via Usp18 aid in the activation of antiviral T- and B-cell responses during viral infection, thus bridging innate and adaptive immune responses [141,142,143]. Moreover, the liver is a major site of macrophage residency and houses Kupffer cells as well as monocyte-derived macrophages [144]. Once more, subset specific functional attributes can be found, as, for example, MerTK^+^ HLA-DR^hi^ macrophages control the resolution of inflammation during acute liver failure via induction of neutrophil apoptosis and clearance [145]. Again, application of scRNAseq techniques aids in further characterization of macrophage subsets, as in human livers two subsets of CD68^+^ macrophages (set 1: Lyz^+^ CSTA^+^ CD74^+^: inflammatory macrophages; subset 2: CD5L^+^ MARCO^+^ VSIG4^+^ C5AR1^+^ KLF4^+^ and others: tolerogenic macrophages) were identified [146]. Similarly, in the heart, macrophages with varying functionalities have been described. Notably, IL-10-producing macrophages have been implicated in diastolic dysfunction, as connexin 43-expressing macrophages contribute to electrical conduction within the myocardium [147,148].

### 5.3. Overarching Macrophage Phenotypes

Aside from organ specific phenotypic attribution, distinct subsets can be detected across various organs. Using scRNAseq analyses, dichotomic LYVE1^hi^ or MHCII^+^ macrophage subsets were previously defined [149]. Another study concluded that this characterization is insufficiently detailed, again using scRNAseq of CD45^+^ CD64^+^ macrophages within the heart, liver, lung, kidney and brain, and thereby revealing the presence of three distinct populations: (i) Timd4^+^ Lyve1^+^ Folr2^+^, (ii) Ccr2^+^ Cd52^+^ S100^+^ MHCII^hi^ and (iii) Trem2^+^ Apoe^+^ Cd14^+^ MHCII^hi^ macrophages. These macrophage subtypes, even though they show similar expression patterns across organs, could be linked to differential functions within each specific organ [150]. It is thus likely that not only expression by itself, but also imprinting via tissue microenvironments, contributes to immune cell phenotypes and function. Indeed, it has recently been suggested that imprinting of macrophages by host tissues might limit plasticity, especially under inflammatory conditions [101]. Impressively, macrophages also respond to remote damage, partially in a tissue-specific fashion. Herein, both macrophage numbers and macrophage responsiveness phenotypes in remote organs are affected differentially by, for example, myocardial infarction, CLP or stroke. This NK-cell-IFNγ-dependent response leads to priming of alveolar macrophages for combatting of bacterial pneumonia [151]. Although ontogeny of tissue-resident macrophages is known to be of yolk sac origin, and organ-detectable macrophage populations contain both pre-populated resident as well as recruited monocyte-derived macrophages, the macrophage landscape is even more complex [152,153,154]. For example, central nervous system macrophages consist of, on the one hand, prenatal progenitors (deriving into meningeal macrophages and microglia), whereas perivascular macrophages derive perinatally from meningeal macrophages dependent on arterial vascular smooth muscle cells and postnatally depend on Irf8 and Mafb, showcasing that tissue-specific niches are crucial for development and maintenance of macrophage subpopulations [155].

## 6. Neutrophil Phenotypes—The Many Faces of a Single Cell

Similar to macrophages, various neutrophil phenotypes have also been described (Table 2). Mechanisms for phenotype changes include activation/deactivation, (reverse) transmigration, reproduction and uptake of foreign (bacterial) material. Neutrophils can be distinguished by various properties, including surface receptor composition, gene expression and density gradient centrifugation characteristics [156]. A common categorization of neutrophil phenotypes similar to the categorization of macrophage subtypes into N0, N1 and N2 subclasses is performed, which has been extensively summarized already [157,158]. Similar to M2 macrophages, CD206 expression is a key feature of N2 neutrophils. Whereas N1 neutrophils have been primarily regarded as anti-tumorigenic and N2 as pro-tumorigenic [159], neutrophil populations appear to be a bit more complex when taking into consideration all phenotypic aspects. Interestingly, CD206^+^ N2 neutrophils also come into play, for example, in inflammatory ischemic damage, such as stroke. Herein, N2 polarization, in a PPARγ-dependent manner, increases neutrophil clearance and resolution of post-stroke inflammation [160]. Additionally, such temporal polarization of neutrophils could also be detected in the heart, following myocardial infarction [161]. Along this line, neutrophil-derived microvesicles indeed display AnxA1-dependent protective characteristics in, for example, the inflamed joint in experimental inflammatory arthritis [162].

Density gradient characterization of neutrophils includes normal-density neutrophils (NDNs), consisting of terminally differentiated and immature neutrophils, and low-density neutrophils (LDNs), which are quite heterogenous, including both pro-inflammatory and immunosuppressive populations and containing myelocytes and metamyelocytes as well as both CD16^+^ CD11b^+^ mature and CD11b^lo^ CD16^lo^ immature neutrophil precursors, although functional distinctions of NDNs and LDNs are being debated [163,164]. Interestingly, LDNs showing CD16^int^ expression are correlated with disease severity, for example, in COVID-19, showing either a potentially functional role or the regulatory mechanisms impacting neutrophil re-production and activation [165]. Characteristically, pathologic challenges impact the bone marrow and splenic neutrophil composition in that C/EBPe-dependent proliferative neutrophil precursors expand [166]. Indeed, in sepsis patients, CD16 immature granulocytes are associated with lymphopenia and immunosuppressive patient characteristics [167]. Not only CD16 ^int^ or immature granulocytes impact lymphocytes, but also CD16^hi^-identifiable subsets (CD16^hi^ CD62L^lo^ CD11c^hi^ CD11b ^hi^) of mature neutrophils can equally impact T-cell functionality. Herein, these neutrophils can effectively hinder T-cell proliferation and responses via a Mac-1/ROS-dependent inflammation-mediated mechanism [168]. This population of mature NET-producing neutrophils can also target other disease categories, including NET targeting of tumor cells, and shows distinct characteristic features, such as reduced adhesion, potentially facilitating cellular translocation [169,170]. Functional relevance with regard to neutrophil subsets has been highlighted in a model of human-LPS-mediated systemic inflammation. In this model, overall neutrophil populations showed reduced levels of common surface receptors, such as CXCR1/2 and TLR4, but increased respiratory burst priming. Nonetheless, CD16(dim)-banded newly produced neutrophils exhibit reduced antimicrobial functionality, hinting at a rather complex time-dependent neutrophil population landscape present during systemic inflammation, possibly contributing to the various stages of hyperinflammation and secondary infection susceptibility [171].

Activation responses do not only include ROS formation, but also other changes in surface receptor compositions and intracellular signaling cascades. Notably, engagement of chemokines or selectins leads to activation of β2-integrins on the neutrophil surface, subsequently exposing an activated phenotype [3]. Additionally, receptor shedding occurs, and this leads to downregulation of L-selectin, which is by itself crucial for subsequent antibacterial and pro-migratory responses [19]. Other surface markers have also been associated with distinct neutrophil functions and phenotypes. One such marker relevant here is the glycoprotein CD177, which is expressed at variable levels in healthy humans [172]. Even though no apparent phenotype is visible in CD177^lo^ or CD1177^hi^ voluntary donors, and CD177 by itself is incapable of signal transmission, this receptor can signal via β2–integrin interaction and thereby mediate neutrophil migration [172,173]. This receptor, a marker for neutrophil activation, was additionally used for correlation with COVID-19 severity and is also linked to protective mechanisms in inflammatory bowel disease [174,175]. Aside from regulative mechanisms affecting CD177 and integrins, other receptors, such as CD66, are mobilized to the neutrophil surface upon activation and consecutively allow binding of ligands (such as galectins) for induction of secondary immune responses, thus showcasing activation-dependent neutrophil phenotypes [157]. Notably, neutrophil phenotype switching is not only traceable due to classical activation at sites of local inflammation, but distinct subpopulations can be identified, as formerly extravasated cells re-enter into the circulation via a process termed reverse migration [176]. In aged mice, these cells, which are characterized, amongst others, by CD54^hi^ CXCR1^lo^ expression, can traffic to the lung and cause endothelial barrier disruption [80,93,177]. Thus, the presence of neutrophil subpopulations is markedly affected by both local and systemic levels of inflammatory mediators and mechanisms of cellular interplay, impacting surface receptor composition as well as intracellular signaling elements, the latter of which includes the safeguard transcription factor PU.1 [178].

Immune cells are communicative cells. Neutrophils show swarm-like behavior in a LTB_4_-dependent manner, but such a swarm response is self-limited via GPCR desensitization, showcasing auto-responsive inflammatory imprinting mechanisms and presence of phenotypically distinct local-site neutrophil swarm subsets [179,180]. Importantly, overarching principles, such as CXCR2-Bmal1-dependent circadian proteome changes occurring in neutrophils, additionally impact the magnitude of neutrophil phenotypes detectable within the body [181]. Application of scRNAseq techniques for bone marrow, blood and spleen indeed corroborated the existence of neutrophil subsets during steady state and infection, highlighting eight distinct phenotypic populations [182].

## 7. Platelets as Immune Cells and Platelet Phenotypes during Inflammation

Platelets are prominently known for their hemostatic potential. Nonetheless, in recent years, their immunologic potential has been brought into focus [39,183]. Indeed, migration, bundling of bacteria and impacting of leukocyte recruitment are just a few inflammatory and immunomodulatory characteristics which make it valid to address these cellular particles as immune cells [39,51,184,185]. Similar to the distinction of neutrophils into low- and normal-density cells, platelets have also been determined by large-heavy (supposedly young) or small-light (supposedly old) characteristics with varying aggregation responses [186,187]. Platelets are commonly distinguished according to their activation state. Activated platelets can feature a high-affinity GPIIb/IIIa receptor conformation, reduced GPIb levels and P-selectin surface mobilization, amongst others, when confronted with various stimuli. Platelets additionally express the glucocorticoid receptor alpha, which, when activated by prednisolone, impacts P2Y12 ADP signaling to inhibit platelet aggregation [188,189]. Platelets are anucleate cellular particles, which partially restrict the capabilities for phenotypic changes. Nevertheless, they contain various mRNAs and—upon activation—synthesize pro-IL-1β protein and IL-1β in a β3-integrin-dependent manner [190]. Platelets overall contain not only cytokines but various chemokines, which can be rapidly released upon activation [191]. Taking into consideration the numerical magnitude of platelets, the effects of platelet activation and immediate chemokine/cytokine release can be detrimental for the host. Platelets can additionally release extracellular vesicles (EVs). This EV release differs according to cellular interplay, platelet stimulation and disease-specific microenvironmental cues [192,193,194,195]. Interestingly, when exposed to stressors such as inflammation, platelet production within the organism itself changes [196,197]. Occurrence of immature platelets in sepsis patients is well-documented and can serve as a predictor for mortality and identification of sepsis patients [198,199,200]. Equally, the immature platelet fraction can aid in identification of inflammatory complications in cardiac surgery patients [201]. Inflammatory changes, such as increased IL-1β levels, indeed favor megakaryocyte rupture, potentially leading to observable changes in platelet numbers and phenotypes [202]. The mode of production as well as the production site might impact the occurrence of distinct platelet populations. Aside from bone-marrow-derived platelets, pulmonary and splenic platelet production have also been reported. Whereas the lung continuously contributes to platelet production, inflammation or changed platelet demand can lead to shifting in platelet production sites with consequent changes in platelet phenotypes [203]. During sepsis, megakaryocyte progenitors are mobilized to the spleen, where they differentiate in an IL-3-dependent manner to fully mature megakaryocytes, which then produce a distinct population of CD40L^hi^ platelets [50]. Aside from such inflammation-induced or immature platelets, aged platelets can also be detected in the circulation. These platelets feature impaired P-selectin mobilization upon thrombin stimulation together with proteome changes linked to increased apoptosis and senescence and reduced spreading and secretion capacities [204,205]. The de-sialylation of these aged platelets results in a feedback loop, stimulating TPO synthesis and thus platelet reproduction, showcasing the interconnection between circulating platelet subpopulations [206,207]. Other approaches to identifying distinct platelet phenotypes or subpopulations involve discovery of eNOS-positive and -negative platelets within human blood, suggesting these two population phenotypes distinctly regulate thrombosis onset [208]. Other phenotypic occurrences include the detection of serotonin-enhanced procoagulant COAT platelets in stroke patients [209,210,211]. Extensive multiparameter phenotyping of platelets can also serve to identify prostacyclin-specific platelet responses [212], or specific human patient cohorts, even in a population of elsewise healthy-appearing humans, identifying six subgroups with distinct platelet phenotypes and reactivity patterns [213]. In addition, combinatorial proteomics and lipidomics studies have enabled deeper characterization of platelet responses and phenotypic subsets [214]. With regard to the COVID-19 pandemic, platelet phenotyping has aided in the identification of disease severity in patient populations [12]. Surprisingly, even though platelets express FPR2 and react to Annexin A1 [215], no examination has been performed to stratify platelets by their pro-inflammatory and pro-resolution potential, as it appears plausible to distinguish between Plat1 (pro-inflammatory) and Plat2 (pro-resolution) platelets, similar to the macrophage and neutrophil characterization, although more detail and comparative studies are still needed (Table 2) [215,216].

**Table 2:** Commonly ascribed immune cell phenotypes.

Cell Type	Phenotype	Mechanism	Characteristics
**Platelets**	Immature platelets	Emergency thrombopoiesis	- High granularity - Increased size
	Sepsis-induced splenic platelets	IL-3-mediated splenic megakaryocyte maturation	- CD40L^hi^
	Lung-derived platelets	S1P-gradient-dependent platelet shedding in pulmonary microvasculature	- Phenotypic distinction so far unclear [217]
	Young vs. old	Life-cycle dependent aging	- Desialylated old platelets - Stimulation of TPO production
	COATed platelets	Dual stimulation with collagen and thrombin	- Phosphatidylserine exposure - High fibrinogen binding
	Post-emperipolesis platelets	Megakaryocyte emperipolesis followed by thrombopoiesis	- Containing neutrophil membrane fragments
	Plat 1 vs. Plat 2	Differing stimuli and microenvironments	- Pro-inflammatory vs. pro-resolution
**Neutrophils**	NDN	Density gradient purification	- Containing mostly mature neutrophils
	LDN	Density gradient purification	- Containing immature neutrophils - Containing neutrophils with elevated CD66b, CD11b
	N1	Increased following TGF-beta blockade	- Immunostimulating - Cytotoxic, tumor-suppressive - Mature phenotype - Short lifecycle [218] - CD11b^lo^
	N2	PPARy-dependent	- Immunosuppressive, - Tumor-permitting, pro-metastatic - CD206^+^ - Increasingly efferocytosed during stroke
	Reverse-migrated neutrophils	LTB4–Neutrophil Elastase-dependent	- Over-activated - CD54^hi^ CXCR1^lo^ - Capable of causing remote injury
	Aged neutrophils	Life-cycle-dependent aging, microbiota-TLR-MyD88-dependent	- CXCR4^hi^
**Macrophages**	M1	LPS-, IFN-dependent	- Pro-inflammatory
	M2 (a, b, c, d)	IL-10, IL-4-dependent	- Anti-inflammatory
	Alveolar	Localization	- Patrolling of airways - Diverse population (including SiglecF^+^ and SiglecF^−^ cells)
	Interstitial	Localization	- Diverse population (including Lyve1^+/−^, MHCII^+/−^, CX3CR1^+/−^ cells) - Localized in interstitium
	Resident	Organ-inherent resident immune cells	- Not mobilized but detectable both under baseline and inflammatory conditions
	Perivascular	Localization vs. organ-specific expression patterns	- Diverse population [219] - Capable of modulating vascular development
	Profibrotic	Increased in pulmonary bleomycin model	- SiglecF^+^ CD11c^+^ MHCII^hi^
	Monocyte derived	Recruitment of monocytes and maturation to macrophages	- CX3CR1^+^ (mouse)-derived - CD16/CD14 (human)-derived
	Splenic macrophages	Anatomic localization	- Marginal metallophilic macrophages - Marginal zone macrophages - Red pulp macrophages

## 8. Regulatory Aspects of DAMPS, PAMPs, Microenvironments and Cellular Interplay

Inflammation can be regarded as the body’s defense reaction to a stimulus, injury or infection which is either well-controlled and adequately triggered, resulting in the successful combating of an invading pathogen, or occurs in excess or under unwanted circumstances, as can be observed in atherosclerosis and rheumatoid arthritis. Pro-inflammatory characteristics are attributed to the notion that a distinct cellular phenotype or response can be perceived as a main driver of inflammation, leading to the putative identification of the main cell type. Distinct tissues contain various chemokine reservoirs, which can be released and differentially control immune cell influx and activation [111,220]. Depending on the site of injury, cause and duration of damage, the cellular as well as chemokine profiles will vary.

Overall, inflammation is associated with the release of a variety of cytokines and chemokines, including TNF, IL-1, IL-6, IL-8, IL-23, CXCL-1, CXCL-2 and procalcitonin, as well as autacoids, neuropeptides, prostanoids and complement factors. Cytokines and chemokines can differentially affect and activate immune cells and likewise contribute to regulation of vascular permeability and immune cell (re-)production. Upon stimulation with IFN and LPS, macrophages, for example, take on the classic M1 pro-inflammatory phenotype. As can be seen by this, engagement of TLR ligands, such as bacterial components including LPS, thus evokes a pro-inflammatory response which can be limited in a TRAF2-dependent, c-Rel, IRF5-dependent manner [221]. This pro-inflammatory polarization of macrophages by TLR4 is additionally controlled by Girdin, which dampens the extent of the pro-inflammatory responses by suppression of NFkB, CREB and p38 MAPK-pathway activation, as Girdin hinders TLR4 receptor dimerization, which is crucially needed for pro-inflammatory signal transduction [222]. Not only bacterial components do lead to pro-inflammatory phenotypes, but also engagement of, for example, calcium depositions within atherosclerotic lesions will trigger TNF secretion and proinflammatory macrophage responses in a PKC-, ERK- and JNK-dependent manner [223]. With regard to regulation of the dynamics of macrophage phenotypes during inflammation, a rapid mobilizable peritoneal population has been described, and this is capable of invading injured tissue in a CD44- and ATP-dependent manner while switching to an AAM phenotype [224], as peritoneal macrophage polarization depends on retinoic acid and GATA6 [225]. Importantly, various transcription factors have been implicated in the maturation and polarization of M1/M2 macrophages, including PU.1 (global enhancer repertoire, but no effect on polarization), STAT1 (M1), IRF5 (M1), STAT6 (M2), IRF4 (M2), C/EBPb (regulating Arg1, IL10 and Mrc1) and PPARγ (M2) [226,227]. Likewise, the repression of histone H3 proteolytic cleavage, which is mediated by CTSG, ELANE and PRTN3, is involved in the maturation of monocytes to fully functional macrophages [228]. Additionally, polarization responses of macrophages depend on the tissue environment and can be exemplary primed by β2-adrenergic responses and the local microenvironment itself [229,230,231]. Only recently, the olfactory receptor Olfr2/OR6A2 was identified in vascular macrophages and shown to be linked via circulating octanal levels to IL-1β release by macrophages, inflammation and stimulation of atherosclerosis [232]. This highlights the complex regulatory interaction of one signal (in this case octanal) leading to the boosting of an additional pro-inflammatory signal (IL-1). Aside from these proinflammatory signals, anti-inflammatory or signals of mixed phenotypic characteristics are also at play. Thus, as IL-4 directs macrophages towards an M2 phenotype, it also limits neutrophil expansion and interferes with CXCR2/4-dependent tissue translocation [233]. An interesting example is represented by IL-12, which plays a somewhat dual role as it contains a subunit as IL-35 (IL-12p35); this demonstrates that various influencing factors are at play for the priming of an inflammatory response. IL-12 can assist in activation of macrophages as well as NK cells, thus displaying properties of a pro-inflammatory cytokine. Nonetheless, in high-blood-pressure settings, the IL-35 subunit of IL-12 reduced M1 macrophages [234,235]. Equally contributing to the notion of diverse regulatory mechanisms, the developmental endothelial locus-1 protein, on the one hand, hinders leukocyte recruitment when secreted by endothelial cells, but, on the other hand, within macrophages, controls their efferocytotic and pro-resolving properties, indicating how cellular and microenvironmental factors do contribute to phenotypic cellular responses [236]. Although it might appear favorable to introduce M2-polarized macrophages into a setting in which a healing or resolution response is needed, this is not feasible in all conditions, as, for example, topical application of M2 macrophages into diabetic wounds does not aid in wound healing or neutrophil clearance [237].

Cytokines can be released by macrophages and surgery as well as other injuries that are associated with peak cytokine levels, depending on the site of the incision/damage [111]. Cytokines and chemokines bind to specific receptors to start cellular activation cascades, which differentially prime immune cell responses. In the lung, macrophages constantly clear the airways in order to guarantee homeostasis [238]. Comparable mechanisms are at play in other organs and environments, such as the peritoneum, where tissue-resident macrophages prevent neutrophil activation in order to maintain tissue homeostasis [239]. Nonetheless, under inflammatory conditions, neutrophils rapidly invade upon bacterial infection or toxic injury [111]. Endothelial cells and neutrophils are activated, allowing the phenomena of neutrophil rolling, adhesion and extravasation in a selectin- and chemokine-dependent manner [40]. DAMPs and PAMPs both prime neutrophils to take on an activated phenotype. Such an inflammatory microenvironment can also be elicited by the release of ATP from necrotic cells, which subsequently activates the Nlrp3 inflammasome via P2rx7 signaling and results in formyl peptide signals, attracting neutrophils that take on proinflammatory chemotactic functions [240]. Herein, interaction of fMLP with its receptor (FPR1) leads to a BTK-dependent pro-inflammatory phenotype switch, resulting in activation of β2-integrins, more specifically Mac-1 [241]. Nonetheless, in human neutrophils, absence of Btk can also be associated with plasma membrane association of Rac2, Mal, PI(3)K and PTKs, leading to a primed activation state, as BTK under baseline conditions interacts with Mal, controlling its cytoplasmic localization [242]. As an additional signaling response, engagement of the chemokine CXCL-1 and binding to its receptor CXCR2 results in activation of integrin LFA-1 in a talin-1, kindlin-3, Rap/Riam and ILK-dependent pathway [3,243]. Binding of a chemokine to its GPCR and subsequent downstream signaling is additionally controlled and self-limited by desensitization occurring in neutrophils in a GRK2-dependent manner, thereby limiting the swarming behavior of these cells when in a proinflammatory state [179]. Aside from activating signaling receptors, inhibitory receptors can also be found within neutrophils. For example, the ITIM receptor PILRα controls integrin activation and neutrophil responses, in which its absence leads to increased neutrophil recruitment, whereas absence of the ITIM receptor LY49Q is associated with overactivation of β2 integrins, resulting in inadequate leukocyte adhesion, preventing transmigration, by this showing partially opposing signaling responses even within similar receptor classes [244,245].

Neutrophils are migratory cells, relying on adequate actin cytoskeleton reorganization and integrin functionality. Crucial for the well-oriented directional response towards differential chemokine gradients is the PTEN pathway, which regulates PI(3)kinase activity, phospholipase A_2_ and p38-dependent migration, impacting bacterial defense and directionality phenotypes of pro-inflammatory neutrophils [246]. Another integrin regulatory element in neutrophils is the myosin light-chain kinase, which, through activation of Pyk2, mediates β2-integrin activation, additionally linking integrins to actin-cytoskeleton-related mechanistic features [247]. Neutrophil activation switching furthermore crucially relies on calcium and potassium channels, such as KV1.3, ORAI and STIM, which all allow for the coordination of calcium signaling and spikes within the cell, impacting all functions ranging from integrin activation to phagocytosis [248,249,250,251]. Involved in the regulation of β2-integrin activation is additionally the downstream regulatory element antagonist modulator (DREAM), which regulates neutrophil responses following TNF stimulation but not fMLP via interaction with A20, a NFkB regulator and IkB kinase [252]. The overall neutrophil responses are tightly controlled by various transcription factors, as shown in a zymosan air pouch model, including RUNX1 and KLF6, being implicated in both neutrophil maturation and recruitment, while RFX2 and RELB are implicated in neutrophil survival; in this experimental setting, RELB, IRF5 and JUNB regulate neutrophil activation and effector functions [253]. An interesting function has been attributed to neutrophils in injury settings; herein, they transport matrix components to a wounded site, leading to scar formation in a kindlin-3-integrin-activation-dependent manner in which heat shock proteins induce integrin heterodimerization, indicating that an activated, supposedly pro-inflammatory phenotype can be functionally rather diverse, and even take on a profile comparable to reparative M2 macrophage features [254]. Phenotypic modulation of neutrophil responses is due to various activation/deactivation mechanisms. Similarly, reverse migration leads to a phenotypic shift in neutrophils. Herein, cells exhibit ICAM1^hi^CXCR1^lo^ expression levels and are primed for executing subsequent, often distant, damage at remote organs in settings of increased vascular permeability [177,255]. This is controlled by LTB_4_, neutrophil elastase, JAM-C and Mac-1 [256]. Notably, such reverse-migration-regulative phenotype responses might also impact secondary organ damage related to UV light damage of the skin, resulting in neutrophil-mediated kidney injury [257].

Finally, cellular interplay affects neutrophil responses and phenotypes. Therein, platelets prime neutrophils for their proinflammatory responses via PSGL-1 interaction with P-Selectin [258]. This priming goes even further as neutrophil GPR35 is activated by platelet-derived serotonin-derivative 5-HIAA, thereby promoting the extravasation of neutrophils [259]. This platelet–neutrophil serotonin interplay is also known to impact myocardial damage by controlling neutrophil degranulation [260]. Looking at platelet–cellular interplay in more detail, platelets via interaction with Tregs also contribute to macrophage polarization in airways during bacterial pneumonia.Platelet-mediated IL-10/TGFβ release leads to an M2-phenotypic priming of macrophages, impacting macrophage efferocytotic capacity and thereby neutrophil clearance from the airways [51]. Platelets by themselves are mainly regarded as taking on activated or inactive phenotypes. Mechanisms underlying these activation responses have been summarized in the past and include engagement of, for example, ADP receptors, thrombin receptors, vWF receptors or collagen receptors [261]. Importantly, focusing on the immunologic aspects of platelets, the migrating and bacterial-bundling capacity of platelets is linked to GPIIb/IIIa activity, and calcium and Myosin-IIa-dependent trailing edge formation [262]. This repositioning ability of platelets additionally aids in an Arp2/3-dependent manner in the maintenance of vascular integrity and surface repair during inflammatory microbleeding, hinting at a sealing phenotype of these migratory-active platelets [263].

## 9. Translational Findings and Immune Phenomics of COVID-19

Laboratory assessment of patients is vital for understanding current health status, disease progression and therapeutic implications. Mass cytometry and single-cell techniques have aided in the acquisition of large-scale clinical and animal study datasets. Cellular phenotyping and assessment of signaling pathways impacting phenotypic priming of immune cells are thus of tremendous interest as they can help us to understand the immunologic landscape of various patient populations, as summarized in Table 3 [264].

Aside from these purely diagnostic purposes, phenotypic assessment has led to advances in the understanding of mechanistic implications and signaling pathways regulating immune hypo- vs. hyper-activation. Targeting of specific populations as well as distinct pathway elements thus appears tempting for therapeutic purposes [272]. For example, local IL-1 administration might aid in the phenotypic priming of alternatively activated macrophages via activation of GATA-3, and timed local administration of platelets could also potentially aid in the shifting of inflammatory cell phenotypes [51,273]. Furthermore, the PAM3-based M2-phenotypic priming of macrophages was used in translational animal models to improve disease course in lupus and inflammatory colitis [274,275]. Likewise, administration of either high-dose dexamethasone or retinoic acid leads to a shifting in macrophage phenotypes towards M2 in a setting of immune thrombocytopenia, as combinatorial administration leads to a sustained response in these patients [276,277]. Active targeting of on–off switches of both inflammation and resolution might aid in the focused guiding of immune cellular subsets to sites of necessity. Exemplary, an agonistic ChemR23 antibody, activating this resolvin E1 receptor, promotes macrophage efferocytosis and aids in the reduction in neutrophil tissue accumulation, stimulating inflammation resolution [278]. While pursuing another pro-resolution pathway, the FPR2/ALX pathway additionally targets neutrophils themselves via Akt and ERK1/2 signaling in sickle cell disease, limiting the pro-NETosis phenotype of neutrophils and aiding in resolution of thrombo-inflammation [279]. Additionally, dietary peculiarities should be considered for switching of phenotypic properties of immune cells. Indeed, a high-salt diet via induction of hyperglucocorticoidism results in reduced bacterial defense capacity of neutrophils [280].

In addition to these translational disease settings and pathomechanistic peculiarities, recent focus was placed on the COVID-19 pandemic, highlighting consequences of an outraged immune system. As mentioned above, specific cellular subsets, such as neutrophil CD16^int^ populations, correlate with hypercoagulation and disease severity in COVID-19 [165]. The Stark/Massberg laboratory used an intriguing approach in assessing phenotypic configurations of immune cells in COVID-19 patients, revealing disturbed neutrophil and platelet activation patterns in the blood of COVID-19 patients, correlating with disease severity [12]. Additionally, other groups have shown, through various techniques, the tremendous impact COVID-19 has on leukocytes and platelets in particular, including IL-6-mediated platelet activation and STAT3 phosphorylation within monocytes and T cells [281,282,283]. As leukocyte and platelet dysregulated functionality is observable, the neutrophil activation marker CD177 can also be used for assessment of COVID-19 severity [174]. Additionally, a CXCL-8/IL-8-dysregulated prothrombotic neutrophil phenotype, favoring degranulation and NETosis, was ascribed to thrombotic complications in COVID-19 [284,285]. Comparative analyses between rheumatoid arthritis and COVID-19 samples revealed similar pro-inflammatory disease patterns regulated via SPP1 and S100A12, leading to activation of proinflammatory CD14^+^ monocytes and PD-L1^+^ neutrophils [286]. Another study reported on the severity-related increase in immature CD16^dim^ neutrophil subsets with increased levels of CD66b, LOX-1 and CD24 and overall changes in granulocyte populations, which are partly restored upon recovery from COVID-19, showing a near complete absence of immature CD16^dim^ neutrophils and normalization of CXCR1, CXCR4 and CD147 levels [287]. McDonald et al. performed a prospective study in COVID-19 ARDS ICU patients, utilizing mass cytometry for single-cell analysis. This work highlights distinct neutrophil phenotypes, identifying a functional priming towards NET release without alteration of other cellular functions, such as ROS production. This phenotype existed independently of dexamethasone treatment, highlighting the necessity of other more accurate therapeutics targeting discrete neutrophil functional phenotypes [288]. Therapeutic consequences of these phenotypic pathway assessments are so far limited but might aid in the differentiation of patient subcategories and sooner or later the targeted intervention with regard to pro-/anti-inflammatory cell populations.

## 10. Outlook and Conclusions

Deeper analyses of distinct immune cell phenotypes are crucial for assessment and stratification of patient populations and disease patterns. Identification of overarching extracellular and intracellular factors contributing to phenotypic re-composition of the immune system will allow for direct targeting of distinct cellular responses for modes of action, such as combating invading pathogens, preventing microvascular leakage or restoring tissue integrity. Due to the large datasets implicated in phenotype assessment, artificial intelligence and machine learning ought to assist in pattern recognition in order to determine novel cellular subsets or behavioral cellular traits [11]. Nonetheless, we deem crucial the determination of functional and clinical relevance of such increasingly detailed cellular patterns and the link of phenotypic features to diseases; in other words, we need to ascertain what is crucial or important, and what is disposable from a pathomechanistic and pharmacological perspective. Additionally, it is important that we determine whether distinct phenotypes are transient or constant or even permanent, or rather simply fluid-phase patterns resulting from maturation steps of prematurely released populations. After all, the holy grail of immune cell phenotype research remains the discovery of specific cues which evoke a cellular response resembling the desired phenotype: for example, a pro-phagocytic macrophage, clearing all neutrophils, without exerting any damaging effects on the surrounding tissue or fibrotic effects. Then, we might be able to switch on/off as needed or trigger another functional type. Lastly, it is tempting to speculate about the pharmacological utility of distinct immune cell populations for therapy. Indeed, the Ley Lab recently demonstrated that disease-specific monocytes control inflammation and mortality in a cystic fibrosis model, hinting that healthy-phenotype monocytes could potentially aid in therapy for such a disease [289].

## Figures and Tables

**Table 1:** Techniques (selection) for cell phenotype assessment.

Type	Technique	Readout	Pro	Con	Example
**Surface receptor analysis**	FACS, Microscopy	Detection and expression levels of surface molecules	Quick, relatively cheap	Incomplete, biased (depending on antibody selection), possibility of confounding effects (dilution, shedding, etc.)	Nicolai et al. [12]
**Proteomics**	MassSpec	Protein content	Complete representation of protein composition	Relatively expensive, machinery needed	Leite et al. [7]
**Transcriptomics**	Sequencing	Gene expression analysis	“Wholistic” perspective on regulatory mechanisms and phenotypes	Expensive, time-consuming	Ballesteros et al. [13]
**Lipidomics**	MassSpec	Lipid mediator composition	Detailed lipid analysis	Expensive, equipment intensive	Peng et al. [14]
**Metabolic profiling**	UHPLC/MS/MS “Metabotype”	Leukocyte specific metabolite analysis	More detailed cellular characterization	Confounder, expensive	Anders et al. [15]
**Single-cell enzyme secretion**	Single-cell multiplex profiling	Leukocyte enzyme secretion phenotyping	Patient characteristic immune secretory signatures	Not routinely available	Zeming et al. [16]
**Functional assays**	Flow chambers, ROS production, ligand binding, …	Functionality in activation assays	Direct functional readout	Not routinely available	Rossaint et al. [17]
**Behavioral landscape**	Integrated phenotype assessment	Integrated multiparametric overview of cellular motion	Generalistic perspective on cellular behavior and functionality	Labor-intensive workup, not routinely available	Crainiciuc et al. [11]

**Table 3:** Examples of applications of immune cell phenotyping.

Disease	Technique	Observation	Publication
**Sero-positive/sero-negative rheumatoid arthritis (RA)**	Single-cell sequencing	Upregulation of CCL13, 18 and MMP3 in circulating myeloid cells; lack of HLA-DRB5 of ACPA− vs. ACPA+ RA patients.	Wu et al. [264]
	Mass cytometry	Identification of increased CD62L^+^ basophil subset in ACPA+ vs. ACPA− patients.	Koppejan et al. [265]
**Multiple sclerosis (MS) phenotyping**	scRNAseq	Compartmentalized immune cell mechanisms and altered expression profiles	Schafflick et al. [6]
**Sepsis phenotyping**	scRNAseq	PBMC assessment reveals PLAC8 and CLU-dependent CD14^+^ IL1R2^hi^HLA-DR^lo^ monocyte discrimination of bacterial sepsis vs. non-sepsis patients with visible expansion in septic patients	Reyes et al. [266]
	scATAC-seq	PBMCs show prognostic value of overall epigenetic heterogeneity (EG-hi: worse survival)	Chen et al. [267]
**Inflammatory bowel disease (IBD)**	scRNAseq	Identification of mucosal IL1B^+^ macrophages and monocytes in IBD vs. control; PBMCs with increased IL-1β^+^ circulating monocytes in active Crohn’s disease vs. ulcerative colitis.	Mitsialis et al. [268]
**COVID-19 phenotyping**	scRNAseq of BAL	Discrimination of disease severity	Wauters et al. [269]
	scRNAseq of Blood	Cellular atlas of blood immune responses during COVID-19, including developing neutrophil population and HLAII downregulation within PBMCs	Wilk et al. [270]
	scRNAseq of cerebrospinal fluid	Dedifferentiated monocytes in CSF in neuro-COVID-19 patients	Heming et al. [271]
